# Entscheidungsarchitektur in der Gemeinschaftsverpflegung: Strategien für eine nachhaltige Ernährung

**DOI:** 10.1007/s00103-025-04107-4

**Published:** 2025-08-05

**Authors:** Julia Meis-Harris, Simone Dohle

**Affiliations:** Forschungslabor für Gesundheits- und Risikokommunikation, Venusberg-Campus 1, Institut für Hausarztmedizin, Gebäude 05, Universitätsklinikum Bonn, 53127 Bonn, Deutschland

**Keywords:** Interventionen, Nudging, Verhaltensänderung, Institutionelle Verpflegungsdienste, Menüdesign, Interventions, Nudging, Behaviour change, Institutional food services, Menu design

## Abstract

Die Gemeinschaftsverpflegung in Deutschland spielt eine zentrale Rolle bei der täglichen Ernährung von Millionen Menschen und hat somit einen erheblichen Einfluss auf Gesundheit und Nachhaltigkeit. Diese Übersichtsarbeit untersucht die Chancen und Herausforderungen der Entscheidungsarchitektur zur Förderung nachhaltiger Ernährungsweisen innerhalb der Gemeinschaftsverpflegung. Im Mittelpunkt stehen 3 Arten von Interventionen, die in diesem Kontext relativ einfach umgesetzt werden können: 1) Default-Interventionen, bei denen eine Auswahloption als Standard angeboten wird; 2) Verfügbarkeitsinterventionen, bei denen das Angebot gesteuert wird; sowie 3) Empfehlungen in Form von Labels oder Logos.

Wir präsentieren aktuelle Forschungsergebnisse, die demonstrieren, wie diese Interventionen dazu beitragen können, positive Ernährungsentscheidungen zu fördern, während gleichzeitig die Wahlfreiheit der Konsumentinnen und Konsumenten gewahrt bleibt. Strategien wie die Etablierung eines fleischlosen Standardmenüs oder die Erhöhung der Verfügbarkeit fleischloser Gerichte zeigen vielversprechende Ergebnisse, während die Wirksamkeit von Ökolabels je nach Verpflegungskontext uneinheitlich bleibt.

Unser Fazit betont die Notwendigkeit weiterer praxisorientierter Studien sowie die Relevanz eines integrativen Ansatzes, der alle relevanten Stakeholder in den Veränderungsprozess einbezieht. Durch eine ausgewogene Kombination dieser Maßnahmen können Verantwortliche einen signifikanten Beitrag zur Entwicklung einer nachhaltigen und zukunftsfähigen Gemeinschaftsverpflegung leisten.

## Abstract (en)

Institutional food services in Germany play a crucial role in the daily nutrition of millions of people and therefore have a substantial impact on both health and sustainability. This narrative review examines the opportunities and challenges of choice architecture to promote sustainable eating habits within institutional food services. The focus is on three types of interventions that are relatively easy to implement in this context: 1) default interventions, where an option is set as the standard; 2) availability interventions, where the range of offerings is controlled; and 3) recommendations in the form of labels or logos.

We present current research findings demonstrating how these interventions can encourage positive dietary choices while maintaining consumers’ freedom of choice. Strategies such as establishing a meat-free default menu or increasing the availability of vegetarian dishes show promising results, whereas the effectiveness of eco-labels varies depending on the catering context.

Our conclusion emphasizes the need for further practical research and highlights the importance of an integrated approach that involves all relevant stakeholders in the change process. Through a balanced combination of these measures, decision-makers can make a significant contribution to the development of sustainable and future-oriented institutional food services.

## Einleitung

Täglich werden Millionen Menschen in Deutschland durch die Gemeinschaftsverpflegung versorgt. Allein in Schulen und Kitas erhalten fast 7 Mio. Kinder täglich ein warmes Mittagessen und Schätzungen zufolge nehmen rund 9 Mio. Arbeitnehmerinnen und Arbeitnehmer täglich Mahlzeiten in Betriebskantinen ein [1]. Auch in Krankenhäusern spielt die Verpflegung eine zentrale Rolle: Jährlich werden über 18 Mio. Patientinnen und Patienten während ihres Aufenthaltes in Krankenhäusern und Rehabilitationskliniken verpflegt [2]. Die Gemeinschaftsverpflegung muss dabei vielfältigen Ansprüchen gerecht werden und auf die Bedürfnisse verschiedener Zielgruppen wie Kinder, Berufstätige oder Seniorinnen und Senioren eingehen. Dabei ist es entscheidend, dass Mahlzeiten nicht nur gesund, sondern auch geschmacklich ansprechend sind, um eine hohe Akzeptanz zu gewährleisten. Gleichzeitig müssen die Angebote wirtschaftlich gestaltet sein, um sowohl für die Anbieterinnen und Anbieter als auch die Verbraucherinnen und Verbraucher erschwinglich zu sein. Die Gemeinschaftsverpflegung hat zudem eine zentrale Bedeutung für die Nachhaltigkeit, da sie täglich eine große Anzahl von Menschen versorgt und somit direkten Einfluss auf den Ressourcenverbrauch, CO_2_-Emissionen und die Lebensmittelverschwendung hat [3–5]. Das Ernährungssystem insgesamt ist weltweit verantwortlich für ein Drittel der globalen Treibhausgasemissionen und stellt somit einen wesentlichen Treiber des Klimawandel dar [6]. In Krankenhäusern hat die Verpflegung, laut einer detaillierten Lebenszyklusanalyse von 33 Schweizer Einrichtungen, die drittgrößten Umweltauswirkungen [7].

Durch gezielte Maßnahmen wie die Förderung regionaler und saisonaler Produkte, die Reduktion von Fleischkonsum und die Minimierung von Abfall kann die Gemeinschaftsverpflegung maßgeblich zu einer nachhaltigen Ernährungsweise beitragen. Zudem hat sie (insbesondere in Schulen und Kitas) Vorbildcharakter und kann Bewusstsein für eine gesunde und nachhaltige Ernährung schaffen. Unter einer *nachhaltigen Ernährung* versteht man laut Welternährungsorganisation (Food and Agriculture Organization; FAO) und Weltgesundheitsorganisation (WHO) solche Ernährungsmuster, die die Gesundheit und das Wohlbefinden von Menschen fördern und die Umwelt nur wenig belasten [8]. Eine nachhaltige Ernährung gewährleistet Ernährungssicherheit, schützt natürliche Ressourcen wie Wasser und Boden, respektiert kulturelle Vielfalt und sollte wirtschaftlich tragfähig und sozial gerecht sein. Ziel einer nachhaltigen Verpflegung ist es, sowohl gegenwärtigen als auch zukünftigen Generationen den Zugang zu gesunder und nachhaltiger Ernährung zu sichern [8]. Obwohl der Aspekt Gesundheit explizit in die Definition einer nachhaltigen Ernährung durch FAO und WHO aufgenommen wurde, kann es zu Konflikten zwischen Nachhaltigkeits- und Gesundheitszielen kommen. Eine nachhaltige Ernährung stimmt nicht immer mit einer gesunden Ernährung überein [9, 10]. Der Fokus des vorliegenden Artikels liegt insbesondere auf der Förderung nachhaltiger Ernährungsweisen. Da jedoch viele der später vorgestellten Interventionen der Entscheidungsarchitektur sowohl für die Förderung gesunder als auch nachhaltiger Ernährungsweisen relevant sind, werden beide Perspektiven berücksichtigt. Im Literaturüberblick in Tab. 1 führen wir dazu neben der Art der Studie auch den jeweiligen thematischen Fokus auf.

**Tab. 1:** Übersicht der zitierten Literatur nach Interventions- und Studienart mit fokussiertem Themenbereich

Interventionsart	Studienart	Publikation	Fokussierter Themenbereich
*Default-Intervention*	Reviews	Ensaff, 2021 [16]	Ernährungsverhalten allgemein
		Cruwys et al., 2015 [28]	Ernährungsverhalten allgemein
	Feldstudien	Campbell-Arvai et al., 2014 [26]	Fleischkonsum
		Lemken et al., 2024 [30]	Fleischkonsum
	Laborstudien	Ghesla et al., 2019 [29]	Spenden für wohltätige Zwecke
	Labor- und Feldstudien	Taufik et al., 2022 [31]	Fleischkonsum
*Verfügbarkeitsinterventionen*	Reviews	Hollands et al., 2019 [33]	Ernährungsverhalten allgemein
		Allan et al., 2017 [34]	Ernährungsverhalten allgemein
		Grech und Allman-Farinelli, 2015 [35]	Ernährungsverhalten allgemein
		Chernev, 2012 [36]	Ernährungsverhalten allgemein
	Feldstudien	Arrazat et al., 2024 [44]	Fleischkonsum
		Visschers und Siegrist, 2015 [54]	Klimafreundliche Mahlzeit (CO_2_-Emission des Gerichts)
		Garnett et al., 2019 [46]	Fleischkonsum
		Spoyalo et al., 2024 [49]	Fleischkonsum
	Laborstudien	Meis-Harris et al., 2024 [32]	Fleischkonsum
		Parizel et al., 2017 [37]	Gemüsekonsum und -geschmack
		Raghoebar et al., 2019 [41]	Fleischkonsum
	Labor- und Feldstudien	Sela et al., 2009 [38]	Fett- und zuckerreduzierte Lebensmittel (Experiment 1a und 1b)
		Van Kleef et al., 2012 [42]	Vollkornbrotkonsum
		Pechey et al., 2022 [47]	Fleischkonsum
*Logos, Labels, Kennzeichnungen*	Reviews	Majer et al., 2022 [51]	Ernährungsverhalten allgemein
		Potter et al., 2021 [52]	Ernährungsverhalten allgemein
	Feldstudien	Lohmann et al., 2022 [53]	Klimafreundliche Mahlzeit (CO_2_-Emission des Gerichts)
		Visschers und Siegrist, 2015 [54]	Klimafreundliche Mahlzeit (CO_2_-Emission des Gerichts)
		Pechey et al., 2022 [55]	Klimafreundliche Mahlzeit (CO_2_-Emission des Gerichts)
		Potter et al., 2021 [52]	Ernährungsverhalten allgemein
		Luick et al., 2025 [56]	Klimafreundliche Mahlzeit (CO_2_-Emission des Gerichts)
		Sonnenberg et al., 2013 [57]	Gesundheitsfördernde Lebensmittelauswahl basierend auf offiziellen Ernährungsempfehlungen
		Thorndike et al., 2012 [58]	Gesundheitsfördernde Lebensmittelauswahl basierend auf offiziellen Ernährungsempfehlungen
		Vanderlee und Hammond, 2014 [59]	Nährstoffgehalt (Kalorien‑, Natrium- und Fettgehalt von Lebensmitteln) plus gesundheitsfördernde Lebensmittel
	Laborstudien	Rramani Dervishi und Dohle, 2025 [60]	Klimafreundliche Mahlzeit (CO_2_-Emission des Gerichts) und Nutriscore
		Meis-Harris et al., 2024 [32]	Fleischkonsum

**Abb. 1:**
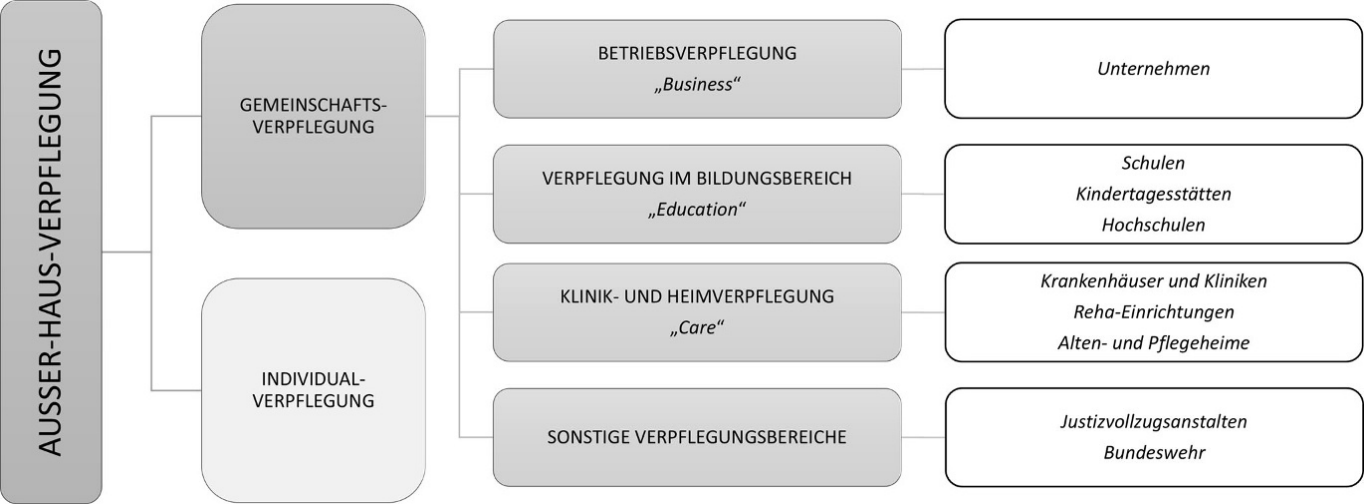
Überblick Gemeinschaftsverpflegung. Abbildung in Anlehnung an Chalupova [62], Teitscheid et al. [63] sowie Pfefferle et al. [4]

Gemeinschaftsverpflegung ist ein wichtiger und wachsender Teil der Außer-Haus-Verpflegung [4]. In Abgrenzung zur Individualverpflegung versteht man darunter die regelmäßige Versorgung bestimmter Personengruppen mit Mahlzeiten und Getränken in gemeinschaftlichen Einrichtungen, wie z. B. Unternehmen, Schulen oder Krankenhäusern (Abb. 1). Die Gemeinschaftsverpflegung wird häufig in verschiedene Segmente unterteilt (z. B. „Business“, „Education“ und „Care“); weitere Unterteilungen sind möglich, beispielsweise in kommerziell und nichtkommerziell wirtschaftende Betriebe [11]. Die Segmente der Gemeinschaftsverpflegung lassen sich weiter nach Einrichtungen und damit nach den jeweiligen Zielgruppen und Rahmenbedingungen der Verpflegung differenzieren. Entsprechend bringt jedes dieser Segmente Möglichkeiten und Herausforderungen mit sich, um Nachhaltigkeit in der Gemeinschaftsverpflegung zu fördern.

Die Deutsche Gesellschaft für Ernährung (DGE) hat Qualitätsstandards für verschiedene Einrichtungen der Gemeinschaftsverpflegung entwickelt, die auch Nachhaltigkeitsaspekte berücksichtigen. Sie stellt zudem detaillierte Leitfäden für die Umsetzung dieser Standards bereit, welche die unterschiedlichen Bedürfnisse der jeweiligen Zielgruppen berücksichtigen. Während für Kliniken beispielsweise ein regenerationsförderndes Verpflegungsangebot im Mittelpunkt steht, geht es bei Kitas und Schulen stärker darum, ein Bewusstsein für gesunde und umweltfreundliche Essgewohnheiten zu schaffen [1, 12]. Die Leitfäden bieten zudem praxisnahe Empfehlungen zur Speiseplanung, Lebensmittelauswahl und -beschaffung sowie zur Integration von Nachhaltigkeitsaspekten in den Alltag der Gemeinschaftsverpflegung.

Verhaltens- und sozialwissenschaftliche Erkenntnisse zeigen, dass nachhaltige Ernährung in der Gemeinschaftsverpflegung entscheidend durch Anpassungen in der Entscheidungsarchitektur gefördert werden kann. Ziel dieser narrativen Übersichtsarbeit ist es, zunächst einen prägnanten Überblick über die wesentlichen Aspekte der Entscheidungsarchitektur im Kontext der Gemeinschaftsverpflegung zu bieten. Anschließend werden wissenschaftliche Studien vorgestellt, die sich mit der Modifikation von Entscheidungsarchitekturen in diesem Bereich befassen. Für die Recherche wurden gängige Datenbanken, insbesondere Web of Science und Google Scholar, unter den Suchbegriffen „Food Choice Architecture“, „Menu Design“, „Nudging“, „Meat“, „Vegetarian“, „Sustainability“ sowie „Sustainable Diet“ genutzt. Dabei wurden insbesondere für die Gemeinschaftsverpflegung relevante Studien nicht systematisch, sondern aufgrund von thematischer Relevanz ausgewählt und extrahiert. Der Fokus des vorliegenden Artikels ist die Förderung nachhaltiger Ernährungsweisen. Am Ende dieser Übersichtsarbeit werden Wissenslücken aufgezeigt und konkrete Handlungsempfehlungen für die Praxis abgeleitet.

## Entscheidungsarchitektur im Kontext der Gemeinschaftsverpflegung

„Entscheidungsarchitektur“ bezeichnet die Art und Weise, wie Auswahlmöglichkeiten dargestellt werden, und spielt eine zentrale Rolle bei der Beeinflussung des Entscheidungsverhaltens [13]. Der Begriff „Nudging“ ist eng mit der Entscheidungsarchitektur verknüpft; ein „Nudge“ (auf Deutsch „Anstoß“ oder „Stupser“) ist eine spezifische Technik oder Intervention innerhalb der Entscheidungsarchitektur, die Menschen dazu ermutigt, eine bestimmte (gesunde oder nachhaltige Ernährungs‑)Entscheidung zu treffen [13]. Dies geschieht häufig durch Anpassungen in der Präsentation, Gestaltung oder Anordnung von Auswahlmöglichkeiten, um bestimmte Entscheidungen wahrscheinlicher, einfacher oder attraktiver zu machen.

Ein zentrales Merkmal von Entscheidungsarchitektur-Interventionen ist ihre Freiwilligkeit [13]. Das bedeutet, dass Nudges die Autonomie der Entscheidungsträgerinnen und -träger bewahren. Bei der Gestaltung von Nudges ist Autonomie somit entscheidend, da sie sicherstellen soll, dass sowohl die Verfügbarkeit von Optionen (*Wahlfreiheit*) als auch die Fähigkeit des Einzelnen, Entscheidungen zu überdenken und zu treffen (*Handlungskompetenz*), erhalten bleiben [14]. In der Gemeinschaftsverpflegung kann das bedeuten, das Angebot an fleischlosen Gerichten zu erhöhen, während gleichzeitig die Möglichkeit erhalten bleibt, fleischhaltige Gerichte zu wählen. Ein „Veggie-Tag“ wird daher nicht als Entscheidungsarchitektur-Intervention angesehen, da durch den Veggie-Tag der Fleischkonsum an bestimmten Tagen ausgeschlossen wird. Nudging ist ein entscheidendes Instrument zur individuellen Verhaltensänderung, dessen Effektivität in einer Vielzahl von Bereichen wie Umwelt, Finanzen, Soziales und insbesondere Ernährung umfassend beschrieben wird [15].

Ernährungsentscheidungen sind vielschichtig, da sie weit mehr umfassen als nur die Auswahl von Lebensmitteln. Die Komplexität bei Ernährungsentscheidungen liegt unter anderem darin begründet, dass bei der Auswahl von Lebensmitteln nicht nur die Produkte selbst, sondern auch physiologische, soziale, kulturelle, psychologische und umweltbezogene Faktoren eine Rolle spielen [16, 17]. Lebensmittelentscheidungen verändern sich darüber hinaus im Laufe der Zeit, beeinflusst durch Alter [18], Generationen, Tages- und Jahreszeit [19, 20] sowie durch Einstellungen [21], Absichten und Gewohnheiten [22]. Wenn Menschen entscheiden, was sie essen, orientieren sie sich oft mehr an automatischen, fest verankerten Mustern als an bewussten, rationalen Überlegungen, die auf Leitlinien und Fakten basieren [23]. Genau hier entfalten Interventionen aus dem Bereich der Entscheidungsarchitektur ihre Wirkung: Sie zielen in der Regel darauf ab, automatische Denk- und Entscheidungsprozesse zu lenken, indem sie die Aufmerksamkeit im Entscheidungsmoment fokussieren und gezielt soziale sowie physiologische Umweltfaktoren gestalten [13, 24]. Ein zentraler Vorteil für Konsumierende besteht darin, dass Nudging nachhaltige Optionen in der Gemeinschaftsverpflegung leichter erkennbar und attraktiver macht – was die Entscheidung für umweltfreundliche Speisen erleichtert, ohne Verzicht zu erfordern.

## Interventionen der Entscheidungsarchitektur zur Förderung einer nachhaltigen Gemeinschaftsverpflegung

Innerhalb der Entscheidungsarchitektur gibt es verschiedene Interventionen, die zur Förderung einer nachhaltigen Gemeinschaftsverpflegung eingesetzt werden können [15]. Im Folgenden legen wir den Fokus auf 3 Arten von Interventionen, die relativ unkompliziert in der Gemeinschaftsverpflegung angewandt werden können: 1) Default-Interventionen, bei denen eine Auswahloption als Standard angeboten wird; 2) Verfügbarkeitsinterventionen, bei denen das Angebot gesteuert wird; und 3) Empfehlungen in Form von Labels oder Logos. Zunächst erläutern wir die zugrunde liegenden Mechanismen der jeweiligen Interventionen, bevor wir auf Praxisbeispiele eingehen.

### Default-Interventionen

Ein Default (im deutschen „Voreinstellung“ oder „Standard“) nutzt die Neigung der Menschen, eine vorgegebene Option beizubehalten, anstatt aktiv eine Änderung vorzunehmen [25]. Gibt es beispielsweise ein Standardmenü, neigen Besucher dazu dieses auszuwählen [26].

Defaults sind wirkungsvoll, da sie dem Prinzip des geringsten Aufwands folgen: Bei den zahlreichen Essensentscheidungen, die wir Tag für Tag fällen müssen, minimiert ein vorgegebenes Standardmenü den kognitiven Aufwand bei der Essensauswahl [16]. Außerdem unterstützt es unsere Tendenz, den Status quo beizubehalten, was in vielen verschiedenen Entscheidungskontexten beobachtet werden kann [27]. Darüber hinaus können Defaults wie ein Standardmenü Aufschluss über die vorherrschenden sozialen Normen geben. Es können somit indirekt Rückschlüsse gezogen werden, welches Essen von den meisten Besucherinnen und Besuchern als angemessen betrachtet wird, was als großer Einflussfaktor auf unser Verhalten gilt [28]. Insgesamt gelten Defaults in der Entscheidungsarchitektur als eine der erfolgreichsten Arten von Nudging-Interventionen [29].

Defaults können auf verschiedene Arten für eine nachhaltige Gemeinschaftsverpflegung eingesetzt werden. Zum einen besteht die Möglichkeit, eine reduzierte Fleischportion als Standardoption anzubieten mit der Möglichkeit, auf Wunsch eine größere Portion anzufordern. Lemken und Kollegen [30] untersuchten diesen Typ des Defaults in der Kantine einer Rehaklinik. Die Ergebnisse zeigten, dass 90,6 % der Besucherinnen und Besucher die kleinere Fleischportion akzeptierten, was verdeutlicht, dass Menschen eher dazu neigen, die vorgeschlagene (in diesem Fall kleinere) Portionsgröße zu akzeptieren, wenn sie aktiv eine Entscheidung gegen die Standardoption treffen müssen. Ein anderer Ansatz besteht darin, ein vollständig fleischfreies Gericht oder Menü als Standardoption anzubieten, mit der Möglichkeit, Fleisch hinzuzubestellen. So entschieden sich beispielsweise Gäste einer Universitätsmensa häufiger für fleischfreie Optionen, wenn das Menü von vornherein vegetarisch war, insbesondere wenn die Fleischgerichte an weniger zugänglichen Stellen platziert waren (z. B. an einer separaten Wand in der Mensa, [26]). Auch durch subtile Veränderungen auf der Speisekarte eines Restaurants können Verbraucherinnen und Verbraucher dazu ermutigt werden, weniger Fleisch zu wählen. Dies gelingt, indem die pflanzliche Option als Standard festgelegt wird, während gleichzeitig die Wahlfreiheit erhalten bleibt, indem alle Optionen, einschließlich der Fleischgerichte, weiterhin auf der Karte verfügbar sind [31]. Defaults können somit auf unterschiedliche Weise individuell an die vorgegebene Gemeinschaftsverpflegung angepasst werden.

### Verfügbarkeitsinterventionen

Eine weitere oft eingesetzte Strategie zur Förderung einer nachhaltigen Gemeinschaftsverpflegung ist die Erhöhung des Angebots bestimmter Gerichte. Wenn beispielsweise in einem Krankenhausspeiseplan nicht mehr nur 1 von 3 wählbaren Gerichten vegetarisch ist, sondern 2 von 3, ist das ein Beispiel einer erhöhten Verfügbarkeit [32].

Ein Cochrane-Review [33] sowie weitere Übersichtsarbeiten [34, 35] bestätigen den positiven Zusammenhang zwischen einem erhöhten Angebot und einem erhöhten Verzehr nachhaltiger und gesunder Lebensmittel. Die psychologischen Mechanismen hinter Verfügbarkeitsinterventionen sind noch nicht vollständig geklärt, dennoch gibt es verschiedene Ansätze dazu, wie Änderungen in der Lebensmittelverfügbarkeit das Verbraucherverhalten beeinflussen könnten. Zum einen steigert eine erhöhte Verfügbarkeit die Wahrscheinlichkeit, dass Verbraucherinnen und Verbraucher Produkte finden, die ihren Bedürfnissen entsprechen [36]. Ein breiteres Angebot kann außerdem Zufriedenheit und Produktpräferenzen erhöhen. Studien zeigen beispielsweise, dass mehr vegetarische Optionen die Vorliebe für Gemüse fördern [37]. Ein größeres Sortiment kann zudem insbesondere Verbraucherinnen und Verbraucher mit einem hohen Bedürfnis nach Vielfalt zufriedenstellen [36, 38]. Schließlich macht die gesteigerte Verfügbarkeit bestimmter Lebensmittel diese nicht nur präsenter, sondern fungiert auch als Hinweis auf soziale Normen, die das Verbraucherverhalten beeinflussen [33, 39–42]. Bei erhöhter Verfügbarkeit vegetarischer Optionen könnten Konsumierende diese als beliebter (deskriptive Normen) und gesellschaftlich akzeptiert (injunktive Normen) wahrnehmen, was die Wahrscheinlichkeit erhöht, dass sie diese wählen [41].

Pechey und Kollegen [40] identifizieren 3 Arten von Verfügbarkeitsinterventionen, die jeweils eine Änderung der folgenden Aspekte betreffen: (1) absolute Verfügbarkeit, (2) relative Verfügbarkeit und (3) kombinierte absolute und relative Verfügbarkeit. Die Änderung der *absoluten Verfügbarkeit* bezieht sich auf die Anpassung der Gesamtanzahl an angebotenen Optionen, wobei die Anteile der Teilmengen unverändert bleiben. Man könnte sich hierzu eine Betriebskantine vorstellen, die normalerweise 6 Menüs anbietet, darunter 2 fleischlose und 4 fleischhaltige. Bei einer Erhöhung der absoluten Verfügbarkeit würde die Kantine insgesamt 10 Menüs anbieten, davon 3 fleischlose und 7 fleischhaltige. Das Verhältnis zwischen fleischlosen und fleischhaltigen Menüs bliebe dabei nahezu gleich. Bei einer Änderung der *relativen Verfügbarkeit* bliebe die Gesamtanzahl der Menüs konstant bei 6, jedoch würden deren Anteile verschoben: Statt 2 gäbe es nun 3 fleischlose und statt 4 nur noch 3 fleischhaltige Menüs. Dadurch würde sich das Verhältnis auf 50 % fleischlos und 50 % fleischhaltig ändern. Die Anpassung der relativen Verfügbarkeit ist in der Gemeinschaftsverpflegung häufig sinnvoller und praktikabler, da sie keine Veränderung der Anzahl der Essensausgaben erfordert.

Sowohl absolute als auch relative Verfügbarkeitsinterventionen können das Wahlverhalten beeinflussen. Jedoch sind relative Verfügbarkeitsinterventionen in der Regel effektiver, da sie die visuelle Wahrnehmung verändern, Hinweise auf soziale Normen bieten und die Wahrscheinlichkeit erhöhen, dass unter dem erhöhten Anteil bestimmter Speisen die persönlich bevorzugte Option enthalten ist [43]. Indem sie den Anteil verschiedener Optionen anpassen, können relative Verfügbarkeitsinterventionen die Lebensmittelauswahl besser in Richtung einer nachhaltigeren Entscheidung lenken.

In der Gemeinschaftsverpflegung werden Verfügbarkeitsinterventionen unterschiedlich eingesetzt. In einer Studie in einer französischen Universitätscafeteria wurde durch eine relative Verfügbarkeitsintervention, die den Anteil vegetarischer Gerichte von 24 % auf 48 % erhöhte, eine Steigerung des Verzehrs von vegetarischen Mahlzeiten um 96 % erzielt [44]. Nicht nur das Wahlverhalten veränderte sich dabei positiv, sondern auch die Menüzufriedenheit stieg leicht an [44]. Die Zufriedenheit bzw. Akzeptanz des Menüangebots ist ein Aspekt, der in der Entscheidungsarchitektur zur Förderung nachhaltiger Verpflegung zurzeit nur sehr selten erhoben wird, für die langfristige Akzeptanz dieser Interventionen jedoch von großer Bedeutung ist [45].

Ähnliche Studien zur Wirksamkeit von Verfügbarkeitsinterventionen im Vereinigten Königreich zeigen ebenfalls positive Einflüsse, berichten jedoch geringere Effektstärken [46, 47]. Garnett und Kollegen [46] erhöhten in 3 Universitätscafeterien den relativen Anteil vegetarischer Hauptgerichte von 25 % auf 50 % und berichten, dass der Anteil der ausgewählten vegetarischen Gerichte durchschnittlich um 61 % stieg. Ein weiteres Feldexperiment zeigt, dass die Wahl vegetarischer Hauptgerichte um 50 % zunahm, nachdem deren Verfügbarkeit in einer britischen Universitätscafeteria von 33 % auf 67 % erhöht wurde [47]. Für die unterschiedlichen Effektstärken in diesen beiden Studien gibt es 2 mögliche Erklärungen: Zum einen unterscheiden sich die Umgebungsfaktoren der französischen und britischen Universitäten, da in Frankreich weniger Alternativen zur Universitätscafeteria vorhanden sind, was die Studierenden stärker an die angebotenen Optionen bindet. Zum anderen könnten die unterschiedlichen Effektstärken auf die konkret angebotenen Hauptgerichte und die Vertrautheit der Teilnehmenden mit den angebotenen Gerichten zurückzuführen sein.

Über die Wirksamkeit von Verfügbarkeitsinterventionen in der deutschen Universitätsverpflegung liegen uns derzeit keine Studien vor. Eine Umfrage unter mehr als 1000 Teilnehmenden in der Universität Mannheim ergab jedoch eine erhebliche Unterstützung seitens der Studierenden und Mitarbeitenden für ein verstärkt pflanzenbasiertes Angebot in der Mensa [48]. Über 80 % der Befragten, die auch Fleisch konsumierten, würden ein solches erweitertes Angebot begrüßen. Die Mehrheit wünschte sich überwiegend vegetarische oder rein pflanzliche Optionen, wobei im Durchschnitt nur 1,5 von 5 gewünschten Gerichten Fleisch oder Fisch enthalten sollten. Diese Ergebnisse verdeutlichen, dass ein erweitertes pflanzenbasiertes Angebot nicht nur akzeptiert ist, sondern sogar stark bevorzugt wird und somit als wirkungsvoller Ansatz zur Förderung nachhaltiger Universitätsstrukturen gelten kann [48].

Studien aus Kanada belegen, dass eine erhöhte Verfügbarkeit nachhaltiger Lebensmittel auch in Krankenhauscafeterien zu einem erhöhten Konsum führt und dass diese Intervention einen stärkeren Einfluss auf die Lebensmittelauswahl hat als die bloße Hervorhebung (Salienz) der nachhaltigen Gerichte [49]. Es zeigte sich, dass sowohl eine Erhöhung als auch eine Verringerung der Verfügbarkeit von nachhaltigen Lebensmitteln die Entscheidungen der Verbraucherinnen und Verbraucher signifikant beeinflusste. Diese Ergebnisse belegen, dass die Erhöhung der Verfügbarkeit von nachhaltigen Lebensmitteloptionen eine effektive Strategie sein kann, um nachhaltige Ernährungsentscheidungen in Krankenhauscafeterien zu fördern.

Im deutschen Kontext zeigt eine Online-Studie zur hypothetischen Essensauswahl in einem Krankenhaus, dass die Erhöhung der relativen Verfügbarkeit von vegetarischen Gerichten im Speiseplan die Auswahl vegetarischer Optionen positiv beeinflusst [32]. Basierend auf dem Menü des Universitätsklinikums Bonn, das täglich 3 verschiedene Gerichte umfasst, weist die Studie darauf hin, dass eine Erhöhung des Anteils vegetarischer Gerichte von einem Drittel auf 2 Drittel zu einer signifikant häufigeren Wahl vegetarischer Speisen führen könnte, ohne die Menüzufriedenheit negativ zu beeinflussen [32]. Die Kombination verschiedener Interventionen – in diesem Fall ein erhöhtes Angebot vegetarischer Gerichte in Verbindung mit einer Essensempfehlung in Form eines Logos – birgt jedoch die Gefahr, die Menüzufriedenheit negativ zu beeinflussen [32].

### Empfehlungen in Form von Logos oder Labels

Empfehlungen, Logos und andere Formen der Lebensmittelkennzeichnung (im Englischen „Labels“) werden häufig eingesetzt, um eine nachhaltige Gemeinschaftsverpflegung zu fördern. Das übergeordnete Ziel besteht darin, Verbraucherinnen und Verbraucher über die ökologischen oder gesundheitlichen Auswirkungen bestimmter Lebensmittel zu informieren. Durch ihre häufig ansprechende und farbige Gestaltung sollen sie die Informationsverarbeitung erleichtern und den kognitiven Aufwand reduzieren. Im Gegensatz zu anderen Interventionen der Entscheidungsarchitektur basieren Labels jedoch auf einer bewussten Informationsverarbeitung. Sie wirken ähnlich wie andere kommunikative Inhalte, bei denen ein Sender einer Rezipientin oder einem Rezipienten eine Aufforderung oder Information (auch als Directive bezeichnet) übermittelt, auf welche die Rezipientin oder der Rezipient reagieren kann [50]. Ein Nutriscore-Label signalisiert beispielsweise, ob ein Produkt für eine ausgewogene Ernährung geeignet ist oder nicht. Doch bleibt die Frage, ob solche Interventionen tatsächlich Ernährungsentscheidungen beeinflussen. Die bisherigen Forschungsergebnisse sind uneinheitlich, sodass eine allgemeine Bewertung der verschiedenen Kennzeichnungssysteme schwierig ist. Letztlich hängt die Wirksamkeit solcher Maßnahmen von mehreren Faktoren ab.

Für die Förderung einer nachhaltigen Gemeinschaftsverpflegung spielen insbesondere Ökolabels (englisch: Ecolabels) eine entscheidende Rolle. Systematische Übersichtsarbeiten bestätigen, dass Ökolabels generell einen positiven Einfluss auf die Kaufentscheidungen für Lebensmittel und Getränke haben [51, 52]. Gleichzeitig betonen sie die Notwendigkeit weiterer Feldstudien, da viele Forschungen unter experimentellen und teils hypothetischen Bedingungen durchgeführt wurden, weswegen die Erkenntnisse nur eingeschränkt auf die Praxis anwendbar sind.

Feldstudien im Bereich der Gemeinschaftsverpflegung zeigen je nach Art der Gemeinschaftsverpflegung unterschiedliche Ergebnisse. In Universitätsmensen führte der Einsatz von Ökolabels zu einer reduzierten Auswahl CO_2_-intensiver Mahlzeiten [53, 54]. In Betriebskantinen ließ sich hingegen kein Effekt von Ökolabels auf die Umweltbilanz der Käufe nachweisen [55]. Diese Ergebnisse wurden auch in einer umfangreichen randomisierten Kontrollstudie in 98 Betriebskantinen im Vereinigten Königreich repliziert. Während der 6‑wöchigen Phase, in der Ökolabels genutzt wurden, gab es keinen signifikanten Unterschied in der Veränderung der gesamten Umweltauswirkung der gekauften warmen Mahlzeiten zwischen den Kontroll- und den Interventionsstandorten [56].

Neben Ökolabels wurde auch untersucht, wie sich Nährwertkennzeichnungen auf Ernährungsentscheidungen auswirken können. Studien in Krankenhauscafeterien haben beispielsweise untersucht, ob Nährwertkennzeichnungen Menschen motivieren könnten, Überlegungen zu gesundheitlichen Aspekten der Mahlzeiten stärker zu berücksichtigen [57–59]. Die Verwendung eines Gesundheitslogos führte beispielsweise zu einer Verringerung des Energie‑, Natrium- und Fettverbrauchs [59]. Ähnlich bewirkte die Einführung von Ampelkennzeichnungssystemen die höhere Auswahl gesünderer Lebensmittel [57, 58]. Experimentelle Laborforschung hat zudem gezeigt, dass sich Nährwertkennzeichnungen auf Lebensmitteln (z. B. Nutriscore) auch auf die wahrgenommene Nachhaltigkeit der Lebensmittel auswirken, was darauf hinweist, dass Nährwertkennzeichnungen auch einen breiteren Einfluss auf Ernährungsentscheidungen haben könnten [60]. Bisher fehlen jedoch Studien, die diesen Zusammenhang in der Praxis zeigen.

## Fazit und Ausblick

Die Ansätze der Entscheidungsarchitektur bieten ein vielversprechendes Instrumentarium, um nachhaltige Ernährungsentscheidungen zu fördern. Restriktive Interventionen, die die Wahlmöglichkeiten einschränken, wie etwa verpflichtende Veggie-Tage, stoßen oft auf Widerstand und können die Akzeptanz von Maßnahmen senken [45]. Im Gegensatz dazu zeigen Entscheidungsarchitektur-Interventionen (wie z. B. Defaults und Verfügbarkeitsinterventionen) häufig positive Effekte, indem sie das Entscheidungsverhalten subtil in eine nachhaltigere Richtung lenken, ohne dabei die individuelle Wahlfreiheit zu beeinträchtigen. Diese Maßnahmen erleichtern Entscheidungsprozesse, indem sie den kognitiven Aufwand reduzieren und auf bereits bestehende Gewohnheiten aufbauen.

Unterstützt werden können diese Maßnahmen unter Umständen durch den Einsatz von Labels und Kennzeichnungen, die Transparenz über die ökologischen und gesundheitlichen Vorteile bestimmter Optionen bieten. Allerdings ist zu beachten, dass die Effektivität von Labels, Logos und Empfehlungen nur in geringen Maßen durch Studien belegt ist und dass die Kombination verschiedener Entscheidungsarchitektur-Interventionen (wie etwa die gleichzeitige Anpassung der Verfügbarkeit und der Einsatz von Labels) auch zu negativen Effekten führen kann. Erste laborbasierte Untersuchungen deuten darauf hin, dass solche Kombinationen in einigen Fällen zu einer verminderten Menüzufriedenheit führen können [32]. Diese Beobachtungen unterstreichen, dass weitere praxisnahe Feldstudien notwendig sind, um die langfristigen Effekte und möglichen unerwünschten Nebenwirkungen differenziert zu beleuchten.

Zudem spiegeln experimentelle Befunde aus kontrollierten Settings nicht automatisch die komplexen Gegebenheiten des Verpflegungsalltags wider, weswegen wir in der vorliegenden Arbeit, wenn möglich, einen besonderen Fokus auf Feldstudien gelegt haben. Für eine bessere Übersicht haben wir in Tab. 1 die verschiedenen Studientypen und zentralen Forschungsinteressen dargestellt. Eine erfolgreiche Umsetzung nachhaltiger Verpflegungskonzepte erfordert einen integrativen und langfristigen Ansatz, bei dem alle relevanten Akteurinnen und Akteure – von Entscheidungsverantwortlichen über Anbietende bis hin zu den konsumierenden Personen – frühzeitig und kontinuierlich in den Veränderungsprozess einbezogen werden. Flexible Anpassungen und regelmäßige Evaluationen sind essenziell, um ökologische, ökonomische und soziale Ziele wirkungsvoll miteinander zu verknüpfen.

Obwohl dieser Beitrag den Schwerpunkt auf Nudging legt, erscheint auch Boosting als ergänzender Ansatz zur Förderung nachhaltiger Ernährung relevant. Beim Boosting handelt es sich um eine verhaltenswissenschaftliche Strategie, die darauf abzielt, die Entscheidungs- und Handlungskompetenzen von Individuen zu stärken (z. B. durch die Vermittlung von Wissen oder Entscheidungsstrategien), sodass sie künftig informierte und eigenständige Entscheidungen treffen können [61]. Allerdings erfordert Boosting in der Regel umfassendere kognitive oder bildungsbezogene Interventionen, die in Settings mit geringer Verweildauer (z. B. Kantinen, Mensen) schwieriger zu implementieren sind [13]. Eine vertiefte Auseinandersetzung mit Boosting-Ansätzen in der Gemeinschaftsverpflegung wäre jedoch ein lohnender Aspekt für zukünftige Forschung.

Insgesamt eröffnen die Ansätze der Entscheidungsarchitektur zahlreiche Chancen, nachhaltige Verpflegungskonzepte erfolgreich zu implementieren. Mit einem ausgewogenen Maßnahmenpaket, das insbesondere auf Defaults und Verfügbarkeitsinterventionen basiert und gegebenenfalls durch sorgfältig geprüfte, zielgruppenspezifische Informationsstrategien ergänzt wird, können Verantwortliche maßgeblich zur Förderung einer gesunden, umweltverträglichen und zukunftsfähigen Gemeinschaftsverpflegung beitragen.
